# Exploring the influence of the Global Fund and the GAVI Alliance on health systems in conflict-affected countries

**DOI:** 10.1186/s13031-015-0031-z

**Published:** 2015-02-02

**Authors:** Preeti Patel, Rachael Cummings, Bayard Roberts

**Affiliations:** Global Health and Security, Department of War Studies, King’s College London, London, UK; Save the Children UK, London, UK; Health Systems & Policy, London School of Hygiene & Tropical Medicine, London, UK

**Keywords:** Global health initiatives, Global Fund, GAVI Alliance, Health systems, Health services, War, Armed conflict

## Abstract

**Background:**

Global Health Initiatives (GHIs) respond to high-impact communicable diseases in resource-poor countries, including health systems support, and are major actors in global health. GHIs could play an important role in countries affected by armed conflict given these countries commonly have weak health systems and a high burden of communicable disease. The aim of this study is to explore the influence of two leading GHIs, the Global Fund and the GAVI Alliance, on the health systems of conflict-affected countries.

**Methods:**

This study used an analytical review approach to identify evidence on the role of the Global Fund and the GAVI Alliance with regards to health systems support to 19 conflict-affected countries. Primary and secondary published and grey literature were used, including country evaluations from the Global Fund and the GAVI Alliance. The WHO heath systems building blocks framework was used for the analysis.

**Results:**

There is a limited evidence-base on the influence of GHIs on health systems of conflict-affected countries. The findings suggest that GHIs are increasingly investing in conflict-affected countries which has helped to rapidly scale up health services, strengthen human resources, improve procurement, and develop guidelines and protocols. Negative influences include distorting priorities within the health system, inequitable financing of disease-specific services over other health services, diverting staff away from more essential health care services, inadequate attention to capacity building, burdensome reporting requirements, and limited flexibility and responsiveness to the contextual challenges of conflict-affected countries.

**Conclusions:**

There is some evidence of increasing engagement of the Global Fund and the GAVI Alliance with health systems in conflict-affected countries, but this engagement should be supported by more context-specific policies and approaches.

Countries affected by armed conflict are of critical global concern in terms of security, development and health. One-and-a-half billion people live in areas affected by armed conflict, fragility or large-scale organized violence. Evidence suggests that conflict-affected countries are significantly worse off than stable resource-poor countries in terms of health outcomes and social determinants of health [1,2]; and are the furthest away from reaching the Millennium Development Goals (MDGs) [3]. Armed conflict damages national health systems through destruction of infrastructure, reducing essential medical supplies, death and displacement of health workers, breakdown of health information systems, and reducing state leadership and governance capacity in all sectors [4-8]. There tends to be heavy reliance on international aid and humanitarian assistance for basic service provision as state capacities are limited in most conflict-affected countries.

Global health initiatives (GHIs) such as the Global Fund (The Global Fund to Fight Aids, Tuberculosis and Malaria), the GAVI Alliance (Global Alliance for Vaccines and Immunisation) and the United States President’s Emergency Plan for AIDS Relief (PEPFAR) emerged at the start of the new millennium in the context of a firm commitment made by international donors to deliver disease-specific health programmes in resource-poor countries [9-11]. GHIs are global health partnerships involving a variety of state and non-state actors such as private sector and civil society organizations and provide a model for financing and implementing disease control programs in low and middle-income countries and regions globally [12]. GHIs provide substantial resources to recipient countries, including conflict-affected countries, to deliver specific health interventions such as vaccines, insecticide treated bed nets, and anti-retroviral therapy. Since 2009, through the work of two major processes of the *High Level Task Force on Innovative International Financing for Health Systems* and the *Maximizing Positive Synergies between Health Systems and Global Health Initiatives*, there has been consensus that optimal health systems are the key to improving health and that GHIs and other donors must move from vertical towards horizontal health financing [13]. While the focus of GHIs work is still on specific communicable diseases, they have in recent years supported national health systems, which directly support their disease specific mandates [10,14-21]. Health system support includes short-term activities that improve services, upgrade facilities or provide salary support, while health system strengthening tends to address longer term, comprehensive approaches that seek to improve multiple aspects such as policies, structures, and regulation [21]. This increased GHI support for health system support has largely been in response to criticisms on the negative effects of GHIs on health systems as a result of their vertical programme focus [22]. There has also been some criticism that GHIs do not have clear policy frameworks for holistic health systems strengthening [23,24]. However, much of the debate and evidence on the role of GHIs and health systems is from stable non-conflict-affected countries. Understanding the interaction between GHIs and health systems support in conflict-affected countries is important as GHIs are major health financers in conflict-affected countries and so have a significant role in strengthening health systems in these countries, even if this focus is principally to support their disease-specific mandates (such as immunisation) [3,25-29]. As most conflict-affected countries have very little government financial resources for health systems, it is important to study the role of GHIs given the substantial influence they may have in health developments and state-building in these countries [24,30].

The aim of this study was to explore the influence of two leading GHIs, the Global Fund and the GAVI Alliance, on the health systems of conflict-affected countries. We focus on these two GHIs specifically as they are two largest GHIs. PEPFAR is not included in this study as it has only very recently begun focussing on a few conflict-affected countries, with most of its traditional focus on stable resource-poor countries, and so did not lend itself to further analysis. The Global Fund and GAVI disburse considerable amounts for health to conflict-affected countries: the Global Fund disburses US$132 million annually for all health activities to conflict-affected countries which is around 25% of all their annual disbursements; while GAVI’s annual disbursements were $38 million to conflict-affected countries which equates to 35% of their total annual disbursements (Table 1, for methodology see [31,32]. Further information on the Global Fund and the GAVI Alliance is given in Table 2. The aim of this study was to explore the influence of the Global Fund and the GAVI Alliance on health systems of conflict-affected countries.

**Table 1:** **Disbursed funding from the Global Fund and GAVI for all health activities to conflict-affected countries between 2005 and 2011**

	**Global Fund**		**GAVI**	
**Country**	**Annual average (US$ millions)**	**Annual average per capita (US$)**	**Annual average (US$ millions)**	**Annual average per capita (US$)**
Afghanistan	1.77	0.07	3.83	0.14
Angola	10.15	0.66	2.38	0.15
Burundi	7.94	0.91	3.26	0.37
Central African Republic	4.93	1.10	0.81	0.18
Chad	3.38	0.35	1.65	0.17
Colombia	3.60	0.09	0.00	0.00
DRC	20.05	0.32	11.98	0.19
Eritrea	5.59	1.05	0.48	0.09
Iraq	1.10	0.04	0.00	0.00
Liberia	6.66	2.08	0.65	0.20
Myanmar	2.75	0.05	0.62	0.01
Nepal	4.21	0.15	1.14	0.04
Sierra Leone	6.42	1.31	0.85	0.17
Somalia	8.46	0.93	0.40	0.04
Sri Lanka	2.02	0.10	1.13	0.06
Sudan	18.97	0.62	5.21	0.17
Timor-Leste	1.11	1.11	0.04	0.04
Uganda	22.49	0.79	3.88	0.14
*Total*	*131.62*	*0.35*	*38.29*	*0.10*

Financial data source is OECD Creditor Reporting Systems (http://stats.oecd.org/Index.aspx?datasetcode=CRS1).

Data are for all health activities and not health systems specifically. US$ data are constant US$ with 2011 as the base year, using CRS deflator rates. Population data from US Census Bureau (http://www.census.gov/en.html). Conflict-affected countries are those classified as in conflict or emerging from conflict based on Uppsala University Conflict Programme Database http://www.pcr.uu.se/research/ucdp/datasets/). For further details on methodology, see http://journals.plos.org/plosmedicine/article?id=10.1371/journal.pmed.1000090.

Syria is not included in the above analysis as the conflict began in 2011.

**Table 2:** **Global Fund and the GAVI Alliance**

**The Global Fund to Fight Aids, Tuberculosis and Malaria (the Global Fund)**	**The GAVI Alliance (GAVI)**
The Global Fund was established in 2002 to significantly increase the resources available to developing countries to address HIV/AIDS, Tuberculosis, and Malaria [33]*.* In recent years, the Global Fund has become one of the major multilateral funders in global health. It channels 82% of the international financing for TB, 50% for malaria, and 21% for HIV/AIDS [34]. The Global Fund began funding for health systems in 2005, and 37% (US$ 362 million) was allocated to health systems support in 2008 [35]. Recipient country ownership has been a key feature of the Global Fund’s policy. The Global Fund does not have an operational presence in the recipient country but it serves as a financial instrument, managing and disbursing resources through an accountable, transparent, independent and technical process which is usually rapid. Each recipient country is responsible for determining its own needs and priorities within the three diseases through the country coordinating mechanism (CCM). The Global Fund gives resources to a principal recipient, which is usually a government institution nominated by the country’s CCM. A local fund agent is also designated to assess a recipient’s capacity to administer funds. As many conflict-affected countries have weak administrative capacity, the role of the local fund agent is in some cases taken by a multilateral organisation such as the United Nations Development Programme (UNDP). Decision-making is based on consultation with a group of diverse stakeholders including national and local governments, NGOs, the private sector and people living with, or affected by, the diseases.	The GAVI Alliance (GAVI) was initiated in January 2000 at the World Economic Forum with the aim of reducing child mortality by providing a rapid delivery of new and improved vaccines for children in low-income countries. GAVI is a public-private partnership which includes: developing and industrialised country governments, research and technical health institutes, industrialised and developing country vaccine industries, civil society organisations, the Gates Foundation and other philanthropy organisations, the WHO, UNICEF and the World Bank Group. In December 2005, the GAVI committed US$500 million for Health System Strengthening for a 5-year period (2006–10), in parallel with its Immunisation Services Support (ISS). This was complemented by an additional US$300 million in 2008 [36]. The GAVI Alliance allocates approximately 15% of its funds for health system support globally [37]. GAVI health system support is aimed at sustaining increased immunization coverage, by providing complementary funding to strengthen health system capacity to provide basic health services in 72 low-income countries [38]. Ministries of Health are invited to use sectoral reviews to identify health systems constraints and to plan responses that will strengthen the health system, and in doing so, improve coverage of immunization and maternal and child health care [36].

## Methods

This study followed an analytical review approach [39]. An analytical review methodology was selected as preliminary research suggested that much of the literature was in the form of quite descriptive evaluation and policy reports which a more restrictive systematic review methodology may exclude.

For the purposes of the review, conflict-affected countries are defined as either currently in armed conflict (intra-state, inter-state or regional) or are in a post-conflict stage. Armed conflict or war refers to violent armed struggle between hostile groups [40]. Post-conflict countries are usually characterised as having: signed a formal peace-agreement; embarked on a process of political transition; increased levels of security; an increased opportunity for peace and reconstruction activities particularly involving international donors. As GAVI and the Global Fund began their health system support in 2005, we focussed on 19 countries which were either in conflict or in a post-conflict phase since 2005 (using criteria from [40,41]). The countries were: Afghanistan, Angola, Burundi, Central African Republic, Chad, Colombia, Democratic Republic of Congo, Eritrea, Iraq, Liberia, Mali, Myanmar, Nepal, Sierra Leone, Somalia, Sri Lanka, South Sudan, Timor Leste and Syria. We also drew on non-country specific literature on fragile states as 75% of fragile states are conflict-affected and these are countries facing severe development challenges and where the government cannot or will not deliver core functions to the majority of its population (such as territorial control, justice, security, capacity to manage public resources, and the delivery of basic services such as health, education, water and sanitation) [42,43].

For the purposes of the review, a health system is broadly defined as the organisations, people and actions whose primary intent is to promote, restore or maintain health. Components include service delivery, human resources, information, products and supplies, vaccines and technology, financing and governance [20].

The review used published and grey literature. Published literature was located through Medline, International Bibliography of the Social Sciences, Web of Knowledge, WorldCat, Social Science Research Network, and the Ingenta bibliographic databases. Examples of search terms entered into the bibliographic databases included: global health initiatives; public-private partnerships; global health partnerships; the Global Fund to Fight Aids, Tuberculosis and Malaria; the Global Fund; the Global Alliance for Vaccines and Immunisation, GAVI; conflict-affected; fragile states; failed states; war; post-conflict; and humanitarian emergencies. No date restrictions were applied, and only documents in the English Language were used.

Similar search terms and criteria were also used to locate relevant grey literature from the internet search engine Google, specialist databases such as ReliefWeb, and the websites of the Global Fund and the GAVI Alliance. Specific reports, factsheets and press materials on GHIs were also obtained through the Global Health Initiatives Network (www.ghinet.org), the WHO’s Maximizing Positive Synergies project (http://www.who.int/healthsystems/GHIsynergies/en/), the Kaiser Family Foundation U.S Global Health Policy website (http://kff.org/global-health-policy/), the Global Fund electronic library (http://www.theglobalfund.org/en/library/, which provided internal and external evaluations of the Fund), and the Global Fund Observer (a newsletter produced by the NGO Aidspan) (http://www.aidspan.org/index.php?page=gfo). These sources included country evaluations for Global Fund and GAVI activities from Burundi, Central African Republic, Chad, Democratic Republic of Congo and Somalia (evaluation reports were reviewed for other conflict-affected countries but they did not include relevant findings).

Our analytical framework was the World Health Organization (WHO)’s building blocks of: health services, health workforce, health information, medical products, vaccines and technology, health financing and leadership and governance within these countries [20]. Data were extracted from the selected evidence based upon these building blocks. This framework enabled us to divide country-level findings from each conflict-affected country by each individual building block. These findings were then compared in the Discussion section using relevant literature on both conflict-affected countries and non-conflict-affected countries to draw out more general findings.

No ethical approval was required for the study as it used a literature review methodology using literature which was already in the public domain.

## Results

The results are based on examination of the primary and secondary evidence identified in the analytical review using findings from primary and secondary published and grey literature, including evaluation reports from conflict-affected countries. The findings are presented below using the WHO health system building blocks framework [20]. In the Discussion, we seek to bring the findings together to highlight commonalities and put them in a context of health systems strengthening more broadly.

### Health services

The evidence highlights significant investment in health services in conflict-affected countries as a result of health system support provided by GHIs. Examples include: antiretroviral therapy (ART) for HIV services in the Democratic Republic of Congo; tuberculosis and malaria services throughout Somalia; and expansion of immunisation programmes in Afghanistan, Liberia and Sierra Leone. However, information on broader health service support was limited. For example, while vaccination coverage was reported to have increased significantly in Liberia as a result of GAVI supported work, information is unavailable on GAVI’s support for health services more broadly in Liberia and any subsequent outcomes [33]. Between 2007 and 2009, Afghanistan reported a six per cent increase in its national DTP3 coverage as a result of health system support from the GAVI Alliance [44], but again little evidence is available on broader health service support and outcomes. GAVI’s health system strengthening support in Burundi increased the number of assisted deliveries as more staff were trained in emergency obstetric support and emergency referral systems were also scaled up [45]. However, estimating the exact contribution that GAVI health system strengthening support interventions have made to this increase is confounded by the fact that Burundi made all prenatal and delivery care free in 2006 [45].

Studies have also raised concerns about the vertical nature of health services supported by GHIs. A study on Somalia questioned the necessity and effectiveness of Global Fund funding for tuberculosis programmes, particularly given the lack of resources for other acute health needs such as reducing the country’s high maternal death rates [27]. Similar concerns were raised with regards to substantial support from the Global Fund to fight HIV/AIDS in Sierra Leone which some in-country stakeholders believed was disproportionately high given the HIV prevalence is less than two per cent [33].

### Health workforce

In most conflict-affected countries, health workers are in short supply and there is a high turnover of health care workers as a result of insufficient salaries in public facilities, poor working conditions and insecurity [46,47]. The limited available evidence suggests that both the Global Fund and GAVI have invested in providing training and support for health workers in conflict-affected countries, with health worker capacity building funds included as a component of Global Fund and GAVI Alliance grants; but it appears they have not sufficiently addressed this issue, particularly with regards to staff retention and motivation which are particularly pertinent in conflict-affected settings [33,48]. The Global Fund and the GAVI Alliance tend to focus on training health workers on technical areas of specific disease focus and have been criticized for dedicating less attention to system-wide topics such as management and capacity building [35,47,49].

### Health information

The limited findings suggest that GHI support for collecting timely health information has been valuable in some conflict-affected countries, where health information tends to be very limited [50,51]. For example, in Somalia, the Global Fund helped establish and maintain the national Health Management Information System that collects monthly data from hospitals in the three zones of the country. In Mozambique, investment from the Global Fund was used to develop and implement an electronic records system in three central hospitals [35]. Liberia and Sierra Leone have also received Global Fund support for collecting health information [35].

### Medical products, vaccines and technologies

GHIs provide substantial resources to conflict-affected countries for vaccines, insecticide treated bed nets, anti-retroviral therapy and other medical products [37]. For example, the Global Fund was the only donor funding anti-retroviral therapy treatment in the Central African Republic [52]. The Somalia national TB programme is entirely dependent on the Global Fund [53]. Funding from GAVI has resulted in increased vaccination coverage in Afghanistan, Liberia, Sierra Leone and several other conflict-affected countries [44].

### Health financing

By mid-2010 the Global Fund had disbursed nearly half (46%) of its overall funding to conflict-affected and fragile countries [33]. It is estimated that 37% of these funds from the Global Fund was directed towards health systems support globally, however it is difficult to ascertain the amounts allocated to the different health system support activities [33,35,54]. The GAVI Alliance allocates approximately 15% of its funds for health system support globally, but it is unclear what proportion of these funds are allocated for health systems supporting conflict-affected countries [37].

Concerns have been raised that the Global Fund’s previous funding model (where upfront allocation is provided for three years for a country) meant there was little scope for adapting to the rapidly changing situations commonly found in conflict-affected countries [43]. The sustainability of GHI financing for health systems has also been raised as a challenge [33]. The Global Fund in particular is under pressure to move quickly with implementation and obtaining rapid results which may undermine long-term health service support [48]. For example, in Central African Republic, concerns were raised about the long-term sustainability of health services should GHI support be reduced or withheld [55].

It was also noted that weak administrative capacity in some conflict-affected countries means that the Global Fund often finds itself in a negative spiral, whereby delays in reporting information delay aid disbursement which leads to delays or reductions in achieving results, which results in poor performance reviews and further reduced spending [43]. As the Global Fund is often the only donor providing lifesaving commodities and related health systems support, such delays lead to stock outs and therefore interruptions in essential treatment [43,52].

GHI funded programmes have also been criticized for a failure to channel key resources to conflict-affected areas or to support refugees or internally displaced persons (IDPs), resulting in potential inequity for key conflict-affected populations [56-58].

### Leadership and Governance

In the Democratic Republic of Congo, GAVI health system strengthening grants are allocated to various civil society organisations that appear to provide strong leadership and management [59]. In Somalia, the Global Fund tuberculosis grant was perceived to provide legitimacy for leadership of civil society resulting in a effective national health programme [60].

Studies have reported that Global Fund grants in fragile states did not perform as well as in stable resource-poor countries [61], with weak governance, corruption and poor leadership consistently identified as constraints in conflict-affected countries [36,51,61]. We could identify no studies providing evidence on specific ways in which GHIs seek to strengthen governance and leadership within the health system.

Concerns over the governance and management arrangements of GHIs themselves in conflict-affected countries were also raised in the literature [61,62]. The Global Fund does not have an in-country presence in any recipient country and potential limitations with this arrangement may be especially acute in conflict-affected countries. In most stable recipient countries, the Global Fund disburses its funds directly to the governments [63]. In countries which lack institutional capacity, such as many affected by conflict, the Global Fund instead uses multilateral or non-governmental organisations to administer grants (e.g. UNICEF in Somalia, UNDP in Syria). As a result, two-thirds of grants in fragile states are administered by multilateral organisations compared to a third of grants in stable resource poor countries [61]. The multilateral organisation is expected to hand over administrative responsibilities to national institutions to strengthen local capacities once sufficient progress and capacity is established. However in many conflict-affected countries this hand over is often problematic, especially if further outbreaks of violence impede capacity building [61,64]. There is a lack of sufficient evidence on whether or not the engagement of international third parties (such as multilateral or non-governmental organisations), as opposed to governments, is the most effective way of health system support in conflict-affected countries [65].

Concerns were also raised in the literature about the responsiveness of GHIs to the contextual challenges of working in conflict-affected situations. For example, South Sudanese health officials spoke of their frustrations about what they perceived as a lack of response from the Global Fund’s in-country partners to the 2014 humanitarian crisis in the country [66]; where looting, closure of health clinics, roadblocks and attacks on health staff has led to the departure of several international partners and interrupted provision and coordination of essential health services supported by the Global Fund [67].

A 2012 external review of the GAVI Alliance also noted its limited capacity to respond to the specific needs of conflict-affected and fragile states, and that GAVI’s rigid performance-based system (where funds are disbursed on the condition that certain targets are met) was not designed to meet the needs of conflict-affected and fragile countries and that a more flexible and context-tailored engagement with such countries was needed [18]. Studies have also noted that a one size fits all performance-based scoring system (as used by the GAVI Alliance and the Global Fund) might not allow for the limited availability and quality in data quality inherent in many conflict-affected countries [43]. The GAVI Alliance has now developed a specific policy for conflict-affected and fragile states in order to be more responsive to their contextual needs, taking a country-by-country approach and sub-dividing countries into those experiencing chronic fragile situations and those experiencing a more acute short term humanitarian emergency [43,68,69]. For countries affected by acute humanitarian emergencies that do not have existing GAVI health system support, the GAVI Alliance will accept an emergency health system strengthening application by the country or by multilateral agencies such as WHO or UNICEF [69]. For countries experiencing chronic long-term conflict, GAVI is endorsing a more flexible and context-specific approach with regard its funding ceilings and channels [69].

## Discussion

To the best of our knowledge, this is the first analytical review on the influence of the Global Fund and the GAVI Alliance on health systems of conflict-affected countries. These GHIs have unquestionably saved or prolonged millions of lives in low and middle-income countries, including conflict-affected countries [70]. Although the evidence is limited, the findings do suggest increasing engagement of both GHIs across all the inter-linked health system components in conflict-affected countries. We should welcome effective and appropriate GHI engagement with conflict-affected countries if it seeks to support health systems and avoids verticalisation and related distortions. Key strengths of the Global Fund and the GAVI Alliance on health systems of conflict-affected countries include: improvements in immunisation coverage, increased financing for health system components such as human resources, improved availability of health information, and greater civil society participation. However, key limitations of their health system support include inequitable financing towards specific diseases, inadequate attention to long-term capacity building, and the lack of specific policies for conflict-affected countries.

Many of the findings from the literature on these GHIs in conflict-affected countries reflect those from studies of stable resource poor countries [9,11]. The literature from stable countries highlights positive effects on health systems including: rapidly scaling up funding; improved access and uptake of health services targeted by the two GHIs; health worker capacity building and training for targeted health services; availability and accuracy of good quality health information related to the targeted interventions; and increasing involvement of civil society organisations [9,11]. Negative effects of GHIs in stable countries include: distorting priorities within the health system such as supplies, equipment, hospital capacity, management and over-site; distorted financing and provision of health services and policies; attrition of the health workforce from the public sector to better funded services targeted by GHIs; and separate supply and parallel information systems which increases bureaucracy, inefficiencies and workload [9,11].

A key issue specific to conflict-affected countries was that GHIs should be more flexible in recognising and responding to the specific health system and contextual factors in conflict-affected countries, particularly related to capacity, security and risk, and how GHI responses should vary during the emergency, post-emergency and reconstruction phases of conflict [71]. The Global Fund appears not to have a specific policy for working in fragile and conflict-affected countries [43,72]. It holds all countries to similar monitoring and evaluation requirements and time frames regardless of their institutional capacity [33], and fails to recognise how conflict-affected countries commonly have very different trajectories of governance and health system capacity [33]. However, it is currently evaluating how to improve overall effectiveness in conflict-affected countries, including how its operating policy can be tailored to different country contexts and capacity levels [43]. This includes exploring the introduction of a more rapid response mechanism, so that, for example, HIV and TB treatment is not interrupted, due to violence and insecurity [43]. In addition, under the Global Fund’s New Funding Model, which was adopted in November 2012, the Fund is committed to a more flexible and context-specific approach, particularly for ‘challenging operating environments’ such as conflict-affected countries. However, it remains unclear how the Fund’s New Funding Model will affect health system strengthening in conflict-affected countries [73]. The GAVI Alliance has recently also followed a more flexible approach to allow it to be more responsive to conflict-affected and fragile country needs, including those in more acute crises. Future research should explore how effectively these new approaches are supporting the overall health system in conflict-affected countries, including at the different stages of crises. This should include whether they are more responsive to the needs of specific conflict-affected populations such as IDPs and refugees [57,74].

The limited available evidence means that it is unclear how responsive GHI activities in conflict-affected countries are to the health system needs of recipient countries [71]. It is also difficult to assess the specific health system effects of the Global Fund and the GAVI Alliance funding given the complexity of actors and influences and contextual constraints. There is also a lack of consensus and clarity on how health system support is to be measured and how such support can be analysed. In-depth country case-studies are needed to investigate and compare how responsive the Global Fund and the GAVI Alliance are to the specific and changing health system needs of acute conflict, early-recovery and post-conflict contexts [10,11]. These should include the way in which contextual factors such as security, overall governance, infrastructure, and technological barriers may influence health systems strengthening. Evidence is also needed to explore these issues at the sub-national level in countries experiencing conflict only in certain regions (for example, Darfur in Sudan or in Eastern Democratic Republic of Congo) so that national-level data do not disguise specific issues in conflict-affected regions. Inter-disciplinary studies drawing on the social and political sciences could be used to explore the influence of GHIs on governance in conflict-affected countries where government capacity, willingness and legitimacy is often challenging [75]. Comparative case-studies of health system capacity and GHI roles in both conflict-affected and non-conflict-affected resource poor countries and settings would also be useful.

The limited evidence also means that it was difficult to determine the extent of health systems support, for example, whether it was circumscribed to those activities that directly support the mandates of the GHIs, or whether they sought to support the health system more broadly. Further research could explore this differentiation further by drawing on work elsewhere on differences between health system strengthening and health system support, as related to particular GHI mandates [21,8].

The limited evidence found in this study focused mostly on health system supply-side issues such as health services, financing, information and medical products. Further research could investigate GHIs’ influence on health system demand-side issues such as barriers to accessing health services (which are particularly pertinent in conflict-affected countries), health-seeking behaviour, health worker motivation, equity, and the quality of health services [21].

Most of the analysis in our paper is based on country evaluations and literature on health systems and GHIs is from African conflict-affected countries, Afghanistan and Syria. This largely reflects the disbursement of conflict and the epidemiological profiles of the conflict-affected countries. However, key information gaps exist on this topic for conflict-affected countries such as Iraq, Nepal and Myanmar.

The limited evidence on GHIs’ influence on conflict-affected countries probably reflects contextual challenges of insecurity, limited management and stewardship capacity, limited operational service delivery capacity, greater challenges with monitoring and evaluation facing GHIs in these settings. In addition, conducting monitoring, evaluation and research in conflict-affected settings is logistically and politically difficult, with little research funding devoted to such settings [76,77].

## Limitations

The main limitation of this study is not using a systematic review approach but this was due to the reasons given above. We also focused only on two GHIs but this was because preliminary analysis suggested that the other major GHIs such as PEPFAR only recently began to engage with conflict-affected countries.

## Conclusion

There is a growing body of literature on the interaction between GHIs and health systems in stable countries. However, this exploratory review suggests there is a very limited evidence-base specifically for conflict-affected countries. The study identified an increasing willingness to invest in conflict-affected countries, rapidly scaling up health system components such as health services, human resources, procurement, guidelines and protocols. However, negative influences included distorting priorities within the health system such as supplies, equipment, hospital capacity, management and over-site, inequitable financing of disease-specific services over other health services, diverting staff from essential health care services to lower priority disease services, inadequate attention to capacity building, and burdensome reporting requirements. Context-specific approaches are required to inform GHI policies and activities in conflict-affected countries given their security, governance and capacity challenges. There appears to be increasing engagement from international donors and agencies, including GHIs, to support health systems in conflict-affected countries, and further research should be conducted to help support this engagement.
